# Three transporters, including the novel Gai1 permease, drive amino acid uptake in *Histoplasma* yeasts

**DOI:** 10.1080/21505594.2024.2438750

**Published:** 2024-12-09

**Authors:** Stephanie C. Ray, Qian Shen, Chad A. Rappleye

**Affiliations:** a Department of Microbiology, Ohio State University, Columbus, OH, USA; b Department of Biology, Rhodes College, Memphis, TN, USA

**Keywords:** *Histoplasma*, pathogenesis, phagosome, amino acid transporter, macrophage

## Abstract

The dimorphic fungus *Histoplasma capsulatum*, which almost exclusively resides within host phagocytic cells during infection, must meet its nutritional needs by scavenging molecules from the phagosome environment. The requirement for gluconeogenesis, but not fatty acid catabolism, for intracellular growth, implicates amino acids as a likely intracellular nutrient source. Consequently, we investigated *Histoplasma* growth on amino acids. Growth assays demonstrated that *Histoplasma* yeasts readily utilize most amino acids as nitrogen sources but only efficiently catabolize glutamine, glutamate, aspartate, proline, isoleucine, and alanine as carbon sources. An amino acid permease-based conserved domain search identified 28 putative amino acid transporters within the *Histoplasma* genome. We characterized the substrate specificities of the major *Histoplasma* amino acid transporters using a *Saccharomyces cerevisiae* heterologous expression system and found that *H. capsulatum* Dip5, Gap3, and a newly described permease, Gai1, comprise most of *Histoplasma*’s amino acid import capacity. *Histoplasma* yeasts deficient in these three transporters are impaired for growth on free amino acids but proliferate within macrophages and remain fully virulent during infection of mice, indicating that free amino acids are not the principal nutrient source within the phagosome to support *Histoplasma* proliferation during infection.

## Introduction

*Histoplasma capsulatum* is a thermally dimorphic fungal pathogen and the causative agent of histoplasmosis, which ranges in clinical presentation from a flulike respiratory illness to widespread disseminated disease [1–3]. As a primary pathogen, *Histoplasma* causes disease in both immunocompromised and in healthy individuals due to a robust virulence program triggered by mammalian body temperature-induced transition to the yeast phase [4]. After inhalation of *Histoplasma*, lung-resident macrophages take up the fungal cells, which grow and replicate within the macrophage phagosome, an environment typically restrictive to microbes. How *Histoplasma* acquires nutrients within this restrictive compartment is poorly understood, with most of the current knowledge pertaining to acquisition of specific micronutrients. Trace metals, such as copper, zinc, and iron, are scavenged by *Histoplasma* using high affinity transporters for copper and zinc and secreted siderophores for iron [5–9]. On the other hand, uracil, tryptophan, and vitamins such as pantothenate and riboflavin are not available within the phagosome, necessitating *de novo* biosynthesis of these compounds by *Histoplasma* yeasts [10–12]. The broader metabolite profile of the macrophage phagosome, especially for fungal pathogens, is generally inferred from transcriptional and genetic studies. For example, phagosomes containing *Candida albicans* and *Cryptococcus neoformans* generally lack sugars, forcing internalized fungi to rely on gluconeogenic carbon sources such as fatty acids and amino acids [13,14]. Gluconeogenesis is also required for *Histoplasma’s* intracellular growth [15]. However, unlike *C. albicans*, which requires the glyoxylate cycle for its virulence [16], fatty acid catabolism is dispensable for *H. capsulatum* during infection, as *Histoplasma* mutants deficient in beta oxidation and the glyoxylate cycle are fully virulent in cultured macrophages and in mice [15]. Since amino acids are additional candidate gluconeogenic substrates inside host cells, we investigated amino acids as potential nitrogen and carbon sources available in the phagosome for intracellular *Histoplasma* yeast.

Amino acid uptake in fungi is carried out by transporters belonging to the well-conserved amino acid-polyamine-organocation (APC) superfamily. In *A. fumigatus, 13* amino acid transporters were upregulated within 12 hours after mouse lung infection [17]. The genomes of *C. neoformans* and *C. albicans* encode at least 9 and 28 amino acid permeases, respectively, and these have been largely characterized based on their homology to the amino acid permeases of *Saccharomyces cerevisiae* [18,19]. Two *C. neoformans* transporters, Aap4 and Aap5, have broad amino acid substrate specificity, and loss of both transporters results in inability to import amino acids at 37°C and attenuated virulence in mice [18]. The amino acid transporters of *C. albicans*, particularly the six general amino acid permease (Gap) homologs, have varying substrate specificities [19–21], and *C. albicans* Gap4 is required for filamentation in response to S-adenosylmethionine [19]. Additionally, in *C. albicans* both Stp2 (an amino acid-responsive transcription factor) and Csh3 (an ER chaperone required for proper folding and trafficking of amino acid transporters) are required for alkalinization of media during growth on amino acids [22,23]. A *C. albicans stp2* mutant was further found to be less virulent in a mouse model of candidemia [23]. More recently, the *GNP* family of amino acid transporters in *Candida* were characterized using toxic amino acid analogs and the transporters shown to be important for fungal survival within and damage to host macrophages, though only minor virulence defects were observed in mice [21]. Undoubtedly, much remains to be understood about the role of host-derived amino acids during fungal infections and which specific amino acids may be available as nutritional sources.

However, most fungal pathogens are largely extracellular during infection. *A. fumigatus* grows as extracellular filaments, and *C. neoformans* and *C. albicans* are only transiently intracellular, having evolved mechanisms for avoiding phagocytosis and inducing their own escape from host macrophages [23–25]. *H. capsulatum*, on the other hand, is almost exclusively intracellular during acute infection, making it a unique model for probing the macrophage phagosomal environment. Here, we examine the adaptation of *Histoplasma* for growth on amino acids by functionally characterizing *Histoplasma’s* ability to import and grow on free amino acids as its carbon or nitrogen source and determine the importance of amino acid import for *Histoplasma* intracellular proliferation and virulence.

## Materials and methods

### *Histoplasma* capsulatum strains and cultivation

The *H. capsulatum* strains used in this study are listed in Table S1A and were derived from the G217B clinical isolate (NAm2 clade, ATCC# 26032). Strains were cultivated as yeasts at 37°C using *Histoplasma*-Macrophage Medium (HMM) or using a defined minimal 3 M-based medium [26] supplemented with carbon and nitrogen sources as described. For general maintenance, strains were plated on HMM media solidified with 0.6% agarose and supplemented with 25 µM FeSO_4_. For growth of uracil auxotrophs, media was supplemented with 100 µg/mL uracil. Yeast liquid cultures were grown at 37°C with continuous shaking (200 rpm). For growth experiments and infections, *Histoplasma* yeast cultures were grown to late exponential growth phase in liquid HMM media prior to use in experiments. Growth was quantified either by measuring culture turbidity via optical density at 595 nm or by enumeration of CFU via plating of serial dilutions on solid HMM media.

### Identification and phylogeny of *Histoplasma* amino acid transporters

Putative *Histoplasma* amino acid transporters were identified by searching the *H. capsulatum* G217B genome for proteins containing the Pfam domains PF00209 (SNF), PF00324 (AA_permease), PF01490 (Aa_trans), PF03222 (Trp_Tyr_perm), PF05525 (Branch_AA_trans), PF13520 (AA_permease_2), or PF13906 (AA_permease_C) using the HMMsearch software (HMMER version 3.3). Proteins matching PF00324, PF01490, and PF13520 were identified, and homologous fungal proteins in other fungi were extracted from the SwissProt protein database for: *Aspergillus fumigatus* Af293 (taxid: 330879), *Aspergillus nidulans* FGSC A4 (taxid: 227321), *Blastomyces dermatitidis* ER-3 (taxid: 559297), *Candida albicans* SC5314 (taxid: 237561), *Coccidioides immitis* RS (taxid: 246410), *Cryptococcus neoformans* var. grubii H99 (taxid: 235443), *Fusarium verticillioides* 7600 (taxid: 334819), *Malassezia globosa* CBS 7966 (taxid: 425265), *Neurospora crassa* OR74A (taxid: 367110), *Saccharomyces cerevisiae* S288C (taxid: 559292), *Schizosaccharomyces pombe* 972 h- (taxid: 284812), *Trichophyton rubrum* CBS 118,892 (taxid: 559305), *Ustilago maydis* (taxid: 5270). Protein sequences were trimmed to contain only the Pfam transporter domain and domain sequences aligned using Clustal (MEGA; version 10.2.4) [27]. Aligned proteins were used to construct a phylogenetic tree to infer orthology using the neighbour-joining method.

### Heterologous expression of *Histoplasma* transporters in *S. cerevisiae*

*Saccharomyces cerevisiae* strain 22Δ10α, which lacks 10 amino acid transporters, was used for heterologous expression studies [28]. *S. cerevisiae* strain 23344c, which retains all amino acid transporters but is otherwise isogenic with 22Δ10α, was used as the wild-type control (both *S. cerevisiae* strains provided by Guillaume Pilot, Virginia Tech). *S. cerevisiae* strains were maintained at 30°C on minimal media containing 2% glucose, 6.7 g/L yeast nitrogen base (YNB) lacking amino acids and ammonium sulfate, 5 g/L ammonium sulfate, and supplemented with 200 mg/L uracil for *ura3* mutant strains. For solid media, 8 g/L agar was included. *S. cerevisiae* strain 22Δ10α was transformed with *URA3*-based *Histoplasma* transporter expression plasmids (listed in Table S1B) using lithium acetate-mediated transformation [29]. *Histoplasma* transporter-encoding genes were amplified by PCR from *H. capsulatum* cDNA and cloned into the pDR196 *S. cerevisiae* constitutive expression vector [30]. Transformants were tested for amino acid import by culture in YNB-glucose medium without amino acids or ammonium sulfate, supplemented with individual amino acids as the nitrogen source in a 96-well microtiter plate. Each amino acid was tested across a range of concentrations up to 4 mM. Wells containing YNB-glucose medium with 4 mM ammonium sulfate were included as positive growth controls. Plates were incubated at 30°C with twice daily agitation at 1000 rpm. Growth was measured daily by culture turbidity via optical density at 595 nm using a BioTek Synergy 2 plate reader until early stationary phase was reached in wells containing wild-type *S. cerevisiae* 23344c at the 4 mM amino acid concentration (approx. 48-72 h, depending on the substrate used). Endpoint growth for each amino acid concentration was determined as the area under the curve across amino acid concentrations (GraphPad Prism software (v9.5.0)) normalized to that of wild-type *S. cerevisiae* 23344c and *S. cerevisiae* 22Δ10α transformed with the empty vector pDR196 (representing 100% and 0% growth, respectively).

### Transcriptional profiling

RNA for reverse transcription quantitative PCR (RT-qPCR) analysis was isolated from log phase wild-type *H. capsulatum* yeasts grown for 24 hours in 3 M media containing 1.5% glucose or 1.5% casamino acids as the carbon source, or from yeasts collected from a 24-hour infection of P388D1 macrophages (MOI 1:1). RNA was released from yeasts via mechanical disruption of yeasts with 0.5 mm-diameter glass beads in TRIzol (Invitrogen) and purified using the Direct-zol RNA Miniprep kit (Zymo Research). For RT-qPCR, cDNA was generated from total RNA using Maxima reverse transcriptase (Thermo Scientific) primed with random pentadecamers. Quantitative PCR was performed using gene-specific primer pairs (Table S2), and products were quantified via SYBR green fluorescence detection (SensiMix SYBR No-ROX Kit, Bioline). Changes in gene expression were quantified relative to the average expression of the constitutive *Histoplasma ACT1, RPS15*, and *TEF1* housekeeping genes using the cycle threshold (Δ*C*_*T*_) method [31].

### Generation of mutant and RNAi *Histoplasma* strains

The transporter-deficient strain lacking the three major transporters was generated by isolation of an 8 base pair deletion of the *GAP3* gene (located at nucleotide 25 of the CDS) using CRISPR/Cas9 [32] in the RNAi sentinel strain, OSU194. The Gai1 and Dip5 transporters were subsequently depleted via RNA interference with a GFP sentinel as previously described (RNAi [33]) using a chimeric RNAi construct consisting of 579 and 771 base pair regions of *GAI1* and *DIP5*, respectively. Knockdown of *DIP5* and *GAI1* was monitored by quantification of the sentinel GFP fluorescence using a modified gel documentation system and ImageJ software (v1.44p) or using a BioTek Synergy 2 plate reader.

### Assays for *Histoplasma* utilization of amino acids and peptides

**Figure 1.**
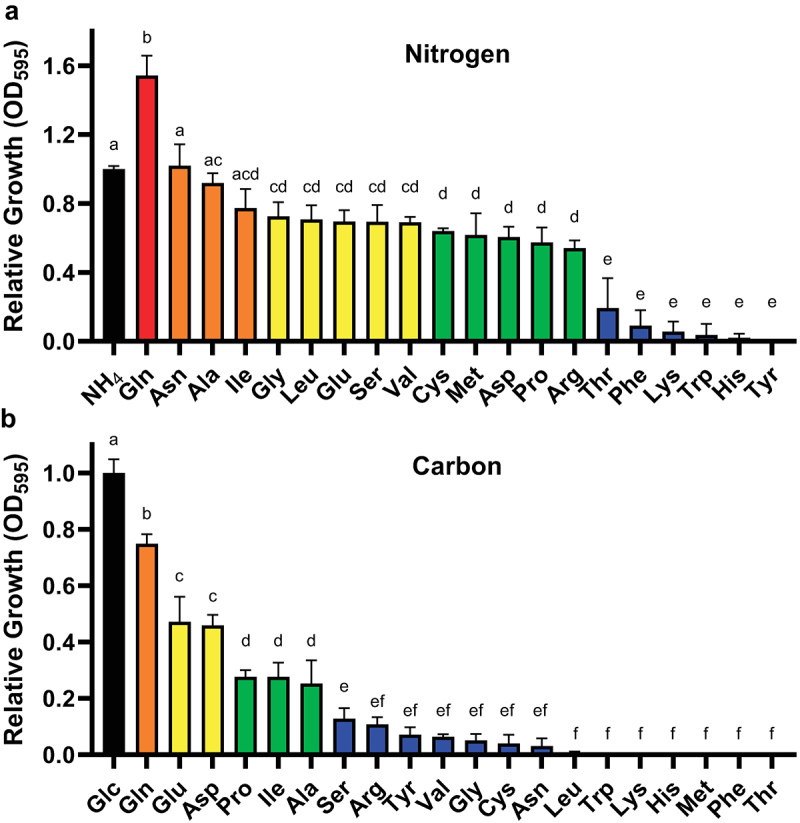
*H. capsulatum* utilizes a range of amino acids as the sole nitrogen or carbon source.

Wild-type *Histoplasma* G217B or the transporter-deficient yeasts were grown to late log phase, rinsed twice, and resuspended in 3 M minimal media with carbon and nitrogen sources at 20 mM and 2 mM concentrations, respectively. To test amino acids as a source of nitrogen, 20 mM of glucose was included in the media (Figure 1a). To test metabolism of free amino acids as a source of carbon, 2 mM of ammonium sulfate was included in the media (Figure 1b). Due to low solubility, tyrosine was tested only at 2 mM concentration in both experiments. To test metabolism of peptides, hemoglobin or gelatin (2%) were incubated at 37°C for 24 h with proteinase K (75 µg/mL, Sigma-Aldrich), trypsin (30 µg/mL, Sigma-Aldrich), or cathepsin D (15 µg/mL, Sigma-Aldrich) and added to 3 M media lacking any carbon or nitrogen in 48-well plates. Growth of yeasts in peptide digests was measured as turbidity (optical density at 595 nm) following incubation at 37°C with continuous shaking at 200 rpm.

Growth of wild type *Histoplasma* yeast in minimal media containing individual amino acids as the sole nitrogen (a) or carbon (b) source. Growth is shown relative to media containing ammonium sulfate (NH_4_) for nitrogen (A) or glucose (Glc) for carbon (B). *Histoplasma* yeast growth was measured via culture optical density at 595 nm after 7 days of growth. Colors correspond to quality of the substrate as a nitrogen (A) or carbon (B) source: red = excellent; orange = good; yellow = fair; green = poor; blue = negligible. Error bars represent standard deviation between 3 biological replicates. Bars annotated with the same letters denote no significant difference between means as determined by one-way ANOVA followed by Tukey’s multiple comparisons test (α = 0.05).

### Macrophage infections

Infection of cultured macrophages used a LacZ-expressing P388D1 murine cell line [34]. Macrophages were cultivated in Ham’s F12 media supplemented with 10% fetal bovine serum (FBS, Sigma) and incubated at 37°C with a 5% CO_2_/95% air atmosphere. Macrophages were infected with *H. capsulatum* yeasts at an MOI (yeasts:macrophages) of 1:3 for intramacrophage CFU enumeration or 1:1 for macrophage killing assays. After 2 hours of infection, non-internalized yeasts were removed by replacing with fresh media. Intracellular proliferation of *H. capsulatum* was determined by lysing macrophages with H_2_O and plating of serial dilutions of the lysate onto solid HMM media for enumeration of colony forming units (CFUs). For macrophage killing assays, surviving macrophages were quantified by measurement of the remaining LacZ activity relative to that of uninfected macrophages [34].

### Murine model of histoplasmosis

To model respiratory and disseminated histoplasmosis, a sublethal inoculum (4×10^4^ *H. capsulatum* yeasts) was introduced intranasally into isoflurane-anesthetized wild type male C57BL/6 mice (Charles River). At 8 days post-infection, lungs and spleens were collected, tissue was homogenized in HMM, and dilutions of the homogenates plated on solid HMM media for CFU enumeration. Animal experiments were performed in compliance with the National Research Council’s Guide for the Care and Use of Laboratory Animals and were approved by the Institutional Animal Care and Use Committee (IACUC) at The Ohio State University [protocol# 2007A0241].

### Statistical analyses

Experimental data were analyzed using GraphPad Prism software (v9.5.0) for determination of statistically significant differences, which are indicated in figures with letters (same-lettered groups are not statistically different) or with asterisks (n.s., not significant; *, *p* < 0.05). Statistical tests used are indicated in figure legends of associated data. Data were collected from 3 biological replicates (*n* = 3) unless otherwise indicated.

## Results

### *Histoplasma* metabolizes select amino acids for growth

Given the importance of gluconeogenesis for *Histoplasma* virulence [15], we investigated which amino acids could support *Histoplasma yeast* growth as potential phagosomal sources of carbon or nitrogen. Utilization of specific amino acids was tested by measuring yeast growth on individual amino acids as the sole nitrogen or carbon source. With glucose as the carbon source, *Histoplasma* yeasts utilized most amino acids for nitrogen, except threonine, phenylalanine, lysine, tryptophan, histidine, and tyrosine (Figure 1a). Glutamine in particular provided an excellent nitrogen source for *Histoplasma* yeasts, consistent with glutamine as a preferred nitrogen source in other fungi [35]. With ammonium as the nitrogen source, a more restricted set of amino acids could supply needed carbon: glutamine, glutamate, aspartate, proline, isoleucine, and alanine (Figure 1b). Interestingly, many amino acids that could supply nitrogen were insufficient sources of carbon, even when provided in 10-fold excess (as carbon versus nitrogen). Tyrosine cannot be fully ruled out as a carbon source, as its solubility required testing at a 10-fold lower concentration compared to the other amino acids. Thus, *Histoplasma* yeasts can metabolize amino acids, but they are limited to select amino acids for nitrogen and particularly carbon.

### The *Histoplasma* genome encodes 28 putative amino acid transporters

**Figure 2.**
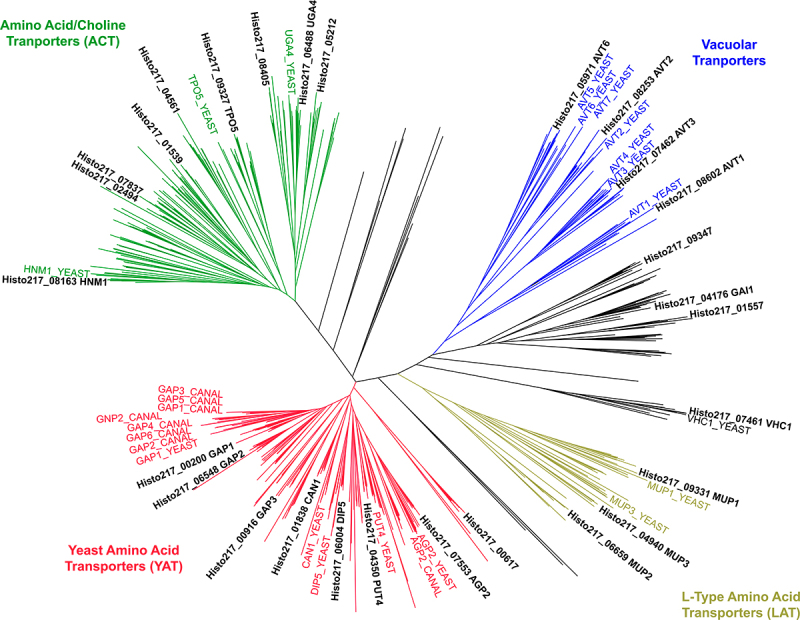
Phylogeny of *Histoplasma’s* 28 predicted amino acid transporters.

For yeast metabolism of amino acids, amino acids must first be imported into the cell via plasma membrane-localized transporters. Therefore, we identified the predicted amino acid transporters encoded in the *Histoplasma* genome. A hidden Markov model (HMM) search of the *Histoplasma* predicted proteome using the Pfam domains PF00324 (AA_permease), PF01490 (Aa_trans), and PF13520 (AA_permease_2) yielded 28 putative amino acid transporters belonging to the amino acid-polyamine-organocation (APC) superfamily of transporters (Table 1). To assign orthology with other fungal amino acid transporters, these proteins and 499 transporter protein sequences from other fungi were aligned and their evolutionary relatedness inferred by construction of a phylogenetic tree (Figure 2). The putative *Histoplasma* transporters separated into 5 major clades, corresponding to groups from the Transporter Classification Database (TCDB): 2.A.3.10 (YAT, Yeast Amino Acid Transporters), 2.A.3.8 (LAT, L-type Amino Acid Transporters), 2.A.3.4 (ACT, Amino Acid/Choline Transporters), and 2.A.18 (AAAP, amino acid/auxin permeases), which includes the vacuolar transporter (AVT) clade and a second less-defined clade with transporters with a range of reported substrate specificities. Assignment of *Histoplasma* transporter designations was based on orthology to experimentally characterized transporters, chiefly from *Saccharomyces cerevisiae*. *Histoplasma* has three transporters corresponding to the general amino acid permeases (Gap transporters) of *S. cerevisiae* which were designated Gap1, Gap2, and Gap3. Within the same Yeast Amino Acid Transporter clade, transporters orthologous to *S. cerevisiae* Can1 (arginine permease), Dip5 (dicarboxylic amino acid permease), Put4 (proline utilization), and Agp2 (plasma membrane sensor) were identified as well as one transporter (Histo217_00617) which did not have an orthologous transporter in the Hemiascomycetes. *Histoplasma*’s L-type amino acid transporters included three proteins designated Mup1, Mup2, and Mup3 due to similarity to *S. cerevisiae* Mup (methionine uptake) proteins. The Amino Acid/Choline Transport clade contained diverse putative transporters, and reliable orthologs were identified for Uga4 (*S. cerevisiae* GABA permease), Tpo5 (putative transporter of polyamines), and Hnm1 (putative transporter of choline, ethanolamine, and carnitine). Multiple *Histoplasma* proteins were found in the Vacuolar Transporter clade that were orthologs to *S. cerevisiae* Avt1, Avt2, Avt3, and Avt6 (amino acid vacuolar transport). In addition, *Histoplasma* has a Vhc1 ortholog (vacuolar protein homologous to cation-chloride cotransporters). Similar to, but not within the Vacuolar Transporter clade is a large group of putative transporter proteins absent in the Hemiascomycetes. One of these we named Gai1 (general amino acid importer 1) based on its deduced transport ability (see below).

Unrooted phylogenetic tree containing 28 predicted H. capsulatum amino acid transporters as well as 499 fungal proteins containing the PF00324 (AA_permease), PF01490 (Aa_trans), or PF13520 (AA_permease_2) Pfam domains. The predicted Histoplasma amino acid transporters separated into 5 broad clades: yeast amino acid transporters (YAT, red), L-type amino acid transporters (LAT, yellow), vacuolar transporters (blue), amino acid/choline tranporters (ACT, green), or unclassified (black). Relevant *S. cerevisiae* (“YEAST”) and C. albicans (“CANAL”) homologs are highlighted in their respective clade colors. A detailed cladogram is at Figshare (https://doi.org/10.6084/m9.figshare.27188757).

**Table 1.** The *Histoplasma* genome encodes 28 putative amino acid transporters.

	*Histoplasma* Gene ID (35)	NCBI Accession #	Designation	Yeast FPKM (35)	Mycelia FPKM (35)	Y:M Ratio	TCDB best fungal hit	e value
Yeast Amino Acid Transporters (YAT)	06004	KAG5291756.1	*DIP5*	375.50	271.87	1.381	2.A.3.10.13	0.00E + 00
	00916	KAG5292284.1	*GAP3*	339.80	10.25	33.151	2.A.3.10.24	1.00E–113
	01838	KAG5298552.1	*CAN1*	94.38	9.83	9.601	2.A.3.10.4	1.00E–175
	04350	KAG5293028.1	*PUT4*	49.34	69.49	0.710	2.A.3.10.17	1.00E–158
	07553	KAG5297634.1	*AGP2*	24.89	63.98	0.389	2.A.3.10.19	1.00E–143
	00617	KAG5291949.1		7.52	111.89	0.067	2.A.3.10.20	1.00E–54
	06548	KAG5288319.1	*GAP2*	4.46	17.95	0.248	2.A.3.10.24	1.00E–154
	00200	KAG5301200.1	*GAP1*	3.77	14.14	0.267	2.A.3.10.24	0.00E + 00
L-Type AA Transporters (LAT)	06659	KAG5287964.1	*MUP2*	44.77	8.56	5.230	2.A.3.8.4	1.00E–40
	09331	KAG5295090.1	*MUP1*	14.29	11.72	1.219	2.A.3.8.4	1.00E–157
	04940	KAG5296624.1	*MUP3*	13.29	5.83	2.280	2.A.3.8.4	1.00E–56
Amino Acid/Choline Transporters (ACT)	09327	KAG5295083.1	*TPO5*	41.81	24.36	1.716	2.A.3.4.2	1.00E–97
	08163	KAG5297000.1	*HNM1*	38.45	21.10	1.822	2.A.3.4.1	0.00E + 00
	02494	KAG5292451.1		21.48	44.07	0.487	2.A.3.4.2	1.00E–67
	07837	KAG5297226.1		18.95	11.51	1.646	2.A.3.4.2	1.00E–82
	04561	KAG5297906.1		15.09	67.46	0.224	2.A.3.4.1	1.00E–37
	05212	KAG5302217.1		9.04	17.50	0.517	2.A.3.4.3	1.00E–75
	01539	KAG5291448.1		8.83	20.11	0.439	2.A.3.4.2	1.00E–69
	08405	KAG5297354.1		5.09	12.80	0.398	2.A.3.4.3	1.00E–80
	06488	KAG5301877.1	*UGA4*	5.04	1.45	3.476	2.A.3.4.3	1.00E–132
Vacuolar Transporters	08602	KAG5287070.1	*AVT1*	65.28	17.22	3.791	2.A.18.5.2	1.00E–118
	07462	KAG5290114.1	*AVT3*	29.58	6.15	4.810	2.A.18.7.3	1.00E–176
	08253	KAG5297544.1	*AVT2*	24.77	23.84	1.039	2.A.18.6.20	1.00E–96
	05971	KAG5291793.1	*AVT6*	20.42	11.84	1.725	2.A.18.6.6	1.00E–94
Unclassified	04176	KAG5292887.1	*GAI1*	619.46	16.61	37.294	2.A.18.4.2	1.00E–81
	07461	KAG5290113.1	*VHC1*	37.32	17.14	2.177	no hits	
	01557	KAG5291464.1		21.61	18.70	1.156	2.A.18.4.2	1.00E–41
	09347	KAG5295107.1		18.71	21.50	0.870	2.A.18.4.2	1.00E–62

**Figure 3.**
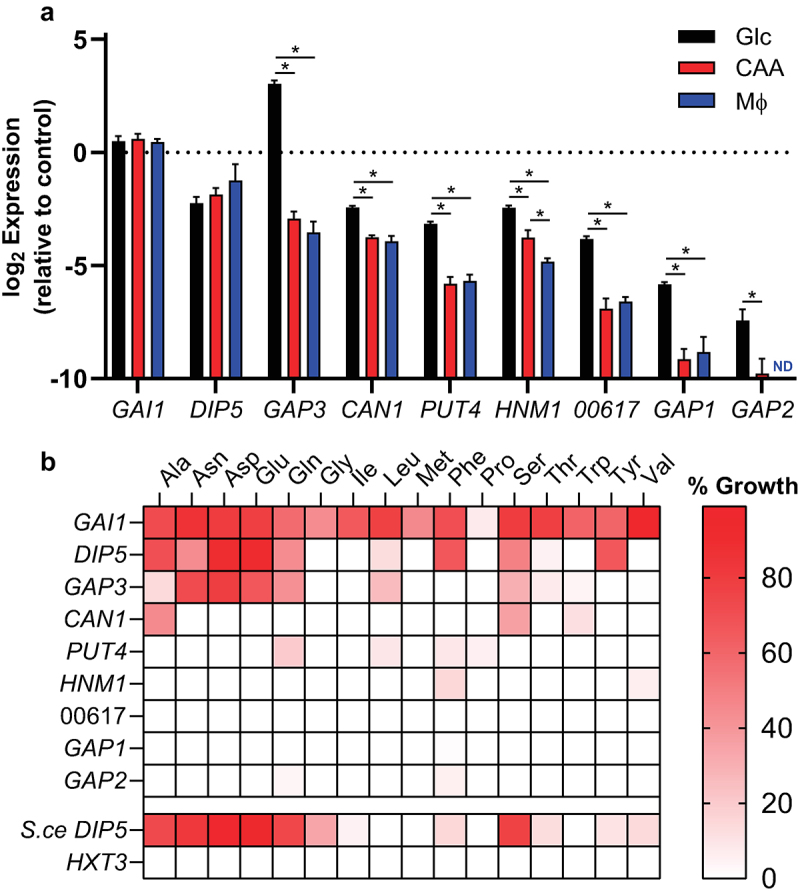
The Gai1, Dip5, and Gap3 permeases comprise most of *Histoplasma*’s amino acid transport capacity.

*Histoplasma* undergoes broad transcriptional rewiring upon transition between the avirulent mycelia and the pathogenic yeast phases. As genes important for *Histoplasma* virulence often show upregulation in the yeast phase [36,37], the relative transcription of each transporter-encoding gene was initially compared using previously published RNAseq datasets collected from *Histoplasma* yeast and mycelia [36]. In yeasts, the genes *DIP5, GAP3*, and *GAI1* showed the overall highest expression (Table 1). *GAI1* and *GAP3* transcription showed enhanced expression in yeasts compared to mycelia (37- and 33-fold, respectively) suggesting functions linked to *Histoplasma* pathogenesis. Some transporter-encoding genes were transcribed at higher levels in mycelia, such as the unannotated genes Histo217_00617 and Histo217_04561. For subsequent transcriptional profiling, we prioritized 9 genes encoding putative plasma membrane-localized transporters: those with overall high or yeast-phase-enriched expression (*GAI1, DIP5*, and *GAP3*) and orthologs of characterized non-vacuolar *Saccharomyces* amino acid transporters (*CAN1*, *PUT4*, *HNM1*, *GAP1*, *GAP3*, and *Histoplasma 00617*). To determine whether these transporters are expressed during macrophage infection, we directly measured their expression in yeasts recovered from infected macrophages and compared gene expression to that of yeasts grown in minimal media with glucose and ammonium sulfate as the carbon and nitrogen sources or yeasts grown in minimal media with casamino acids as the carbon/nitrogen source. Seven genes showed significant upregulation in minimal glucose/ammonium media compared to growth on amino acids or inside macrophages (Figure 3a). Of these, *GAP3* was the most upregulated in glucose (approximately 65-fold). Notably, for all genes except *HNM1* (which had slightly higher expression in amino acid-containing media versus intracellular), there was no significant difference in expression between growth on amino acids versus residence within macrophages, suggesting the macrophage phagosome is most similar to an environment with amino acids rather than hexoses [13,15]. The *GAI1* and *DIP5* genes showed no regulation but consistent high overall expression in yeasts.

(a) Relative gene expression of *H. capsulatum* amino acid transporters from yeasts grown in minimal media containing glucose and ammonium sulfate (“Glc,” black bars), casamino acids (“CAA,” red bars), or during intracellular growth in cultured macrophages (“MΦ,” blue bars). Gene expression is shown relative to the average expression of three *Histoplasma* housekeeping genes (*ACT1*, *RPS15*, and *TEF1*; dotted horizontal line). Error bars represent standard deviation for 3 biological replicates. Asterisks denote statistically significant expression differences as determined via Student’s t test (*, *p* < 0.05). Pairs lacking asterisks were not significantly different. ND = not detected. (b) Growth of the amino acid permease-deficient *S. cerevisiae* strain 22Δ10α on individual amino acids following transformation with heterologously expressed *Histoplasma* genes encoding amino acid transporters, as well as the native *S. cerevisiae* Dip5 transporter (*S.ce DIP5*) and the *H. capsulatum* hexose transporter-encoding gene *HXT3*. Growth is normalized for each amino acid the growth of wild-type *S. cerevisiae* (100%) and the transporter-deficient strain 22Δ10α (0%). Growth data on each amino acid is the average of 3 biological replicates.

### *Histoplasma* Gap3, Dip5, and Gai1 provide the majority of amino acid import

To investigate the substrate specificities of *Histoplasma*’s putative amino acid transporters, their ability to provide amino acid import to an amino acid-transporter-deficient *S. cerevisiae* strain was tested. *Histoplasma* genes encoding the transporters were heterologously expressed in *S. cerevisiae* strain 22Δ10α, which lacks 10 amino acid transporters and is unable to import amino acids [28]. Transport of individual amino acids was inferred by the ability of each *Histoplasma* gene to confer growth to the *S. cerevisiae* yeasts when provided single amino acids as the sole nitrogen source (Figure 3b). Growth was measured in a gradient of amino acid concentrations and normalized to growth of wild type *S. cerevisiae* on each amino acid substrate to account for any differences in *Saccharomyces* metabolic capacity. Zero percent growth was defined as growth of the transporter deficient strain (22Δ10α) transformed with an empty vector. As an approximation of the substrate specificity of the *Histoplasma* transporters, the area under the curve of growth across all concentrations was determined once early stationary phase was reached in the wild type strain at the highest amino acid concentration. As cysteine, histidine, and lysine cannot be utilized by *S. cerevisiae* as nitrogen sources, these amino acids were unable to be tested as growth substrates [28,38]. Arginine also could not be tested as it is readily taken up even in the transporter-deficient *S. cerevisiae* strain [28]. Heterologous expression of *Histoplasma GAI1* conferred growth on all tested amino acids, making it the most promiscuous transporter of those tested (Figure 3b). *DIP5* showed a more narrow substrate range, with aspartate and glutamate being the most readily imported, followed by phenylalanine, tyrosine, alanine, serine, asparagine, glutamine, and leucine. *GAP3*, despite its homology to general amino acid permeases, showed a similar substrate specificity to *DIP5*, with aspartate, asparagine, glutamate, and glutamine supporting *S. cerevisiae* growth, along with serine, leucine, alanine, threonine, tryptophan, and valine to a much lesser extent. The remaining genes tested were extremely limited in their ability to facilitate growth of *Saccharomyces* on amino acids, both in terms of the number of amino acids transported as well as the degree of transport. While Can1 has been reported as a basic amino acid permease in *S. cerevisiae* and *C. albicans* [39,40], the *Histoplasma* Can1 transporter facilitated *S. cerevisiae* growth on alanine, serine, and tryptophan, but only marginally. Other *Histoplasma* transporters provided less than 50% relative growth on few amino acids (Figure 3b). Despite their homology to general amino acid permeases, the *Histoplasma* Gap2 and Gap1 transporters provided little amino acid import and growth to *S. cerevisiae*. *Histoplasma* Gap1 (also referred to as HcCyn1) has previously been demonstrated to confer cystine transport to *S. cerevisiae* [41], highlighting the possibility of other substrates for these transporters. Overall, *GAI1, DIP5*, and *GAP3* were the most effective *Histoplasma* transporters of amino acids in this heterologous expression system.

To validate that the observed amino acid transport was permease-specific, we tested growth of *S. cerevisiae* expressing the *Histoplasma HXT3* gene, which is predicted to transport glucose. As expected, no difference in growth on amino acids was observed between the *HXT3-*expressing strain and *S. cerevisiae* transformed with the empty vector control. As a positive control, the native *S. cerevisiae DIP5* gene (*S.ce DIP5*) was expressed and resulted in transport of dicarboxylic amino acids, consistent with what has been reported in the literature [39].

### Loss of Gap3, Dip5, and Gai1 impairs *Histoplasma* growth on free amino acids

**Figure 4.**
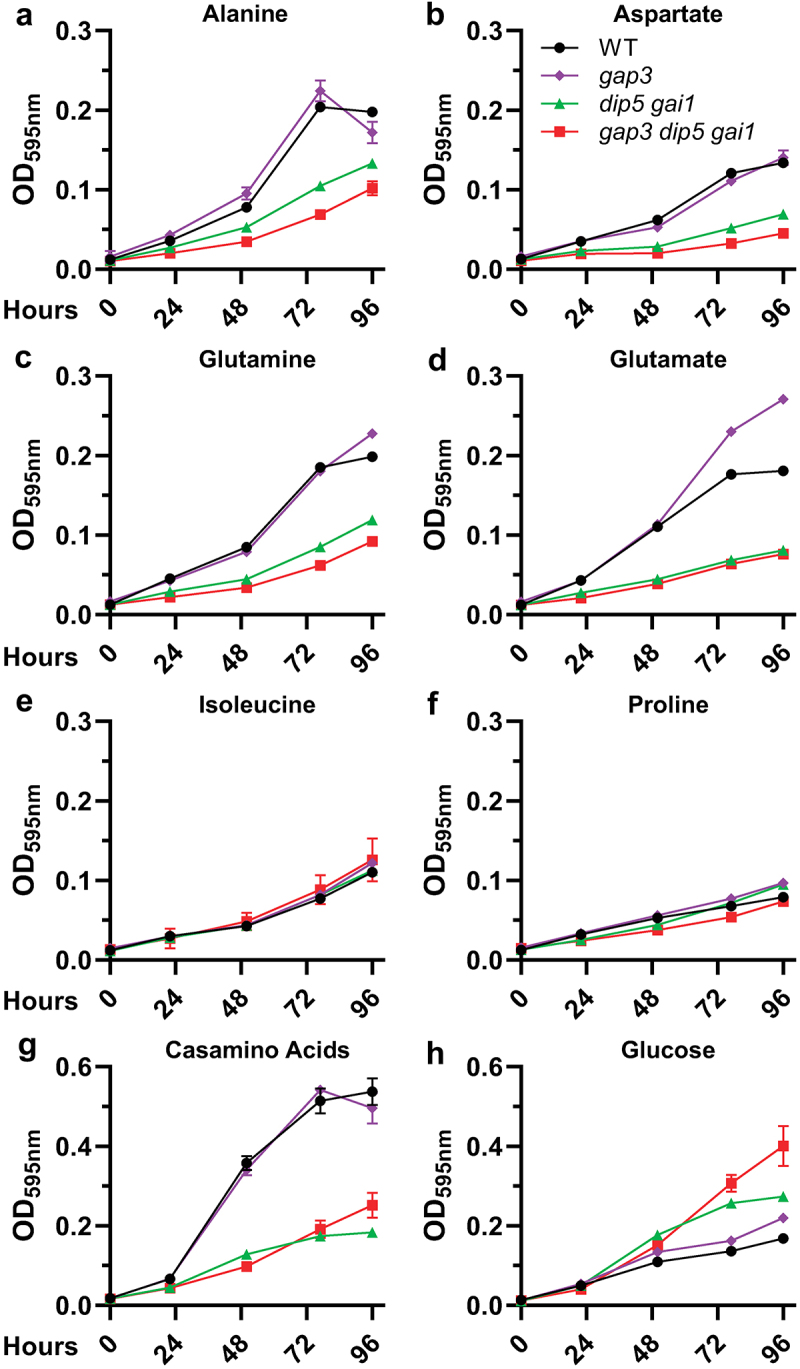
A Gap3 Dip5 Gai1 triple-transporter deficient strain of *Histoplasma* grows poorly on most amino acids.

As *GAP3, DIP5*, and *GAI1* are the most highly expressed amino acid transporter genes in *Histoplasma* yeast, and given the overlap between their transported substrates when expressed in *Saccharomyces* with the profile of amino acids that *Histoplasma* can metabolize (Figure 1), we tested whether loss of these transporters prevented *Histoplasma* yeast growth on amino acids. Given the potential redundancy of the transporters (Figure 3b), we created a strain deficient in all three transporters via a combination of CRISPR/Cas9-based gene disruption (*GAP3*) and RNA interference (RNAi; *DIP5* and *GAI1*). This triple transporter-deficient strain, along with the *gap3* single deletion strain and a *DIP5/GAI1* double-deficient strain, were tested for growth on individual amino acids capable of supporting growth as both the carbon and nitrogen source (Figure 1) as an indication of amino acid uptake and utilization. Compared to wild-type *Histoplasma* yeasts, the triple transporter-deficient strain grew poorly in media with alanine, aspartate, glutamine, or glutamate (Figure 4a–d). The triple-transporter-deficient strain grew as well as wild type on both proline and isoleucine, although wild type itself showed poor growth on these amino acids (Figure 4e,f). Most importantly, compared to wild-type yeasts, the *Histoplasma* transporter-deficient strain showed a marked reduction (78% reduced) in growth rate on casamino acids, an acid hydrolysate of casein containing a mixture of all free amino acids (Figure 4g). Of the three transporters tested, Gap3 contributed the least to the observed phenotypes, as deletion of Gap3 alone had no effect on growth compared to wild type yeasts, save for a slight increase in final density with glutamate (Figure 4d). Depletion of Dip5 and Gai1 gave an intermediate phenotype compared to the triple deficient strain only when grown on alanine, aspartate, and glutamine, but showed a similarly severe reduction in growth rate to the triple deficient strain on casamino acids (83% lower than that of wild-type), suggesting Dip5 and Gai1 are the predominant amino acid transporters in *Histoplasma* yeasts.

Growth of wild type (“WT,” black circles), gap3 mutant (“*gap3*,” purple diamonds), DIP5/GAI1-deficient (“*dip5 gai1*,” green triangles), and triple deficient (“*gap3 dip5 gai1,”* red squares) *Histoplasma* yeasts on alanine (a), aspartate (b), glutamine (c), glutamate (d), isoleucine (e), proline (f), casamino acids (g), or glucose and ammonium sulfate (H) as the carbon and nitrogen source(s). Growth was measured as yeast culture optical density at 595 nm over time (hours, x-axis). Error bars represent standard deviation among 3 biological replicates.

**Figure 5.**
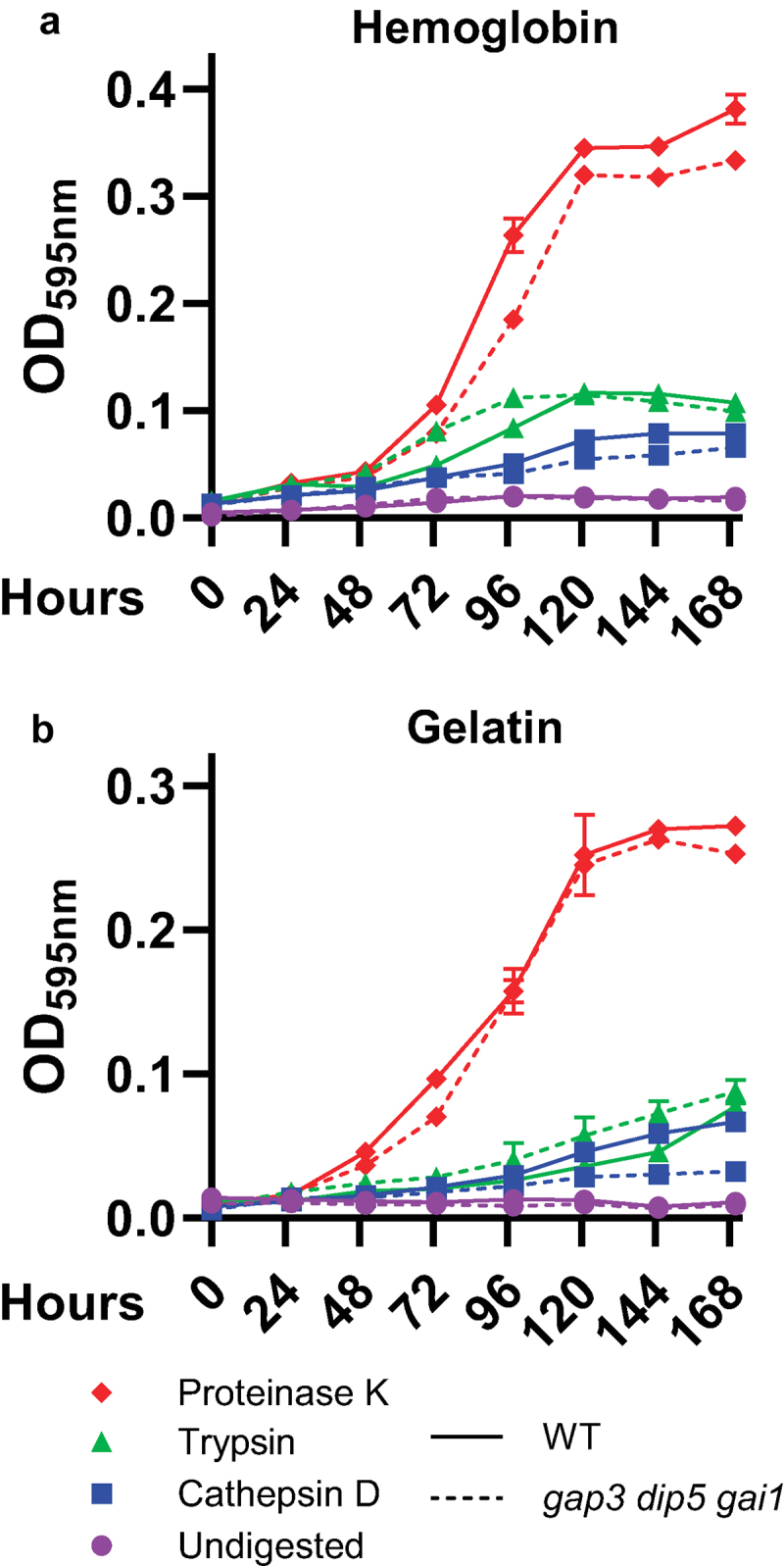
Loss of amino acid transport does not impair growth on digested protein.

During infection, proteins and peptides could provide a source of amino acids for intracellular proliferation of *Histoplasma* yeasts. While the strain of *H. capsulatum* yeast used in this study is not known to secrete proteases, *Histoplasma* has the capacity to grow on short peptides, with more extensive digestion of proteins corresponding with increased growth of the fungus [15,42,43]. To test whether depletion of *Histoplasma GAP3, DIP5*, and *GAI1* amino acid transporters impaired utilization of peptides (i.e. digested proteins), we grew *Histoplasma* yeast in minimal media containing either hemoglobin or gelatin as the sole carbon and nitrogen source, as well as hemoglobin or gelatin digested with cathepsin D (a phagosomal proteinase), trypsin, or proteinase K (Figure 5). Consistent with previously published data, neither wild type *Histoplasma* nor the permease-deficient strain was able to grow on undigested protein [15]. Digestion of proteins into peptides facilitated growth of *Histoplasma* yeasts, and production of shorter peptides (as would be expected from treatment with proteinase K, which has broader protein cleavage specificity) was associated with better *Histoplasma* growth on both protein substrates. However, only slight differences in growth were observed between the wild type and the triple amino acid transporter-deficient strain on these peptide-containing media (Figure 5a–b) consistent with the Gap3, Dip5, and Gai1 transporters being specific for free amino acids. Wild-type yeasts grew slightly better than permease-deficient yeasts on cathepsin D-treated gelatin after 4 days of growth (Figure 5b), but overall growth on this substrate was extremely poor, and this difference was not mirrored in the cathepsin D-treated hemoglobin condition. Therefore, while depletion of Gap3, Dip5, and Gai1 impairs growth on free amino acids, these transporters are not necessary for import of peptide substrates.

Growth of wild type (solid lines) *Histoplasma*yeast and a strain deficient in the Gap3, Dip5, and Gai1 transporters (dashed lines) in media containing 1% hemoglobin (a) or gelatin (b) which had been pre-treated with proteinase K (red diamonds), trypsin (green triangles), cathepsin D (blue squares), or left undigested (purple circles). Growth was measured as yeast culture optical density at 595 nm over time (hours, x-axis). Error bars represent standard deviation between 3 biological replicates.

Intriguingly, the *GAP3/DIP5/GAI1*-deficient yeasts consistently reached saturation more rapidly than wild type *Histoplasma* yeast when grown on minimal glucose medium (Figure 4h). As all *Histoplasma* strains were maintained on rich media containing both glucose and amino acids, this may reflect adaptation of the triple permease-deficient strain to media components other than amino acids, therefore priming this strain for growth on glucose-NH_4_ medium as suggested by the shorter lag before exponential growth in minimal glucose.

### *Histoplasma* yeast virulence does not require the Gap3, Gai1, or Dip5 transporters

**Figure 6.**
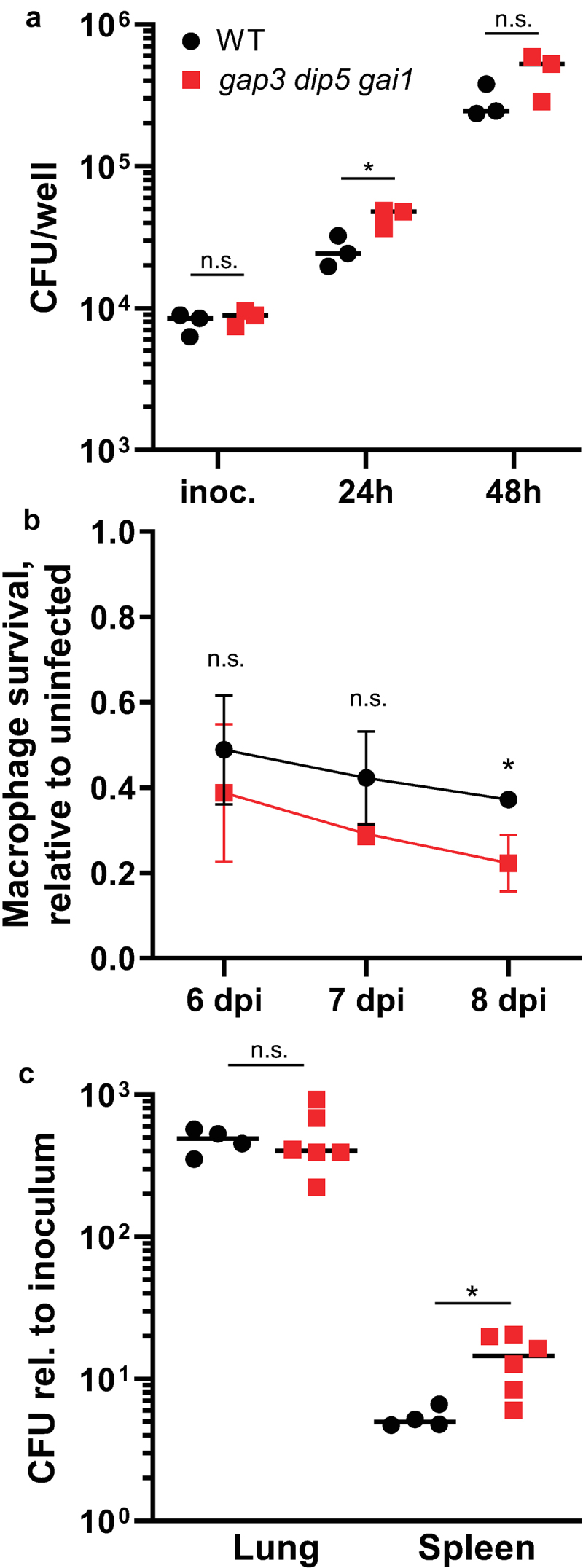
Amino acid transport-deficient *Histoplasma* yeasts are fully virulent in macrophages and in vivo.

As previous work suggests that amino acid metabolism supports growth of *Histoplasma* yeasts within the macrophage phagosome [15], we tested whether amino acid import by yeasts is thus required for *Histoplasma* virulence by measuring the proliferation of *Histoplasma* yeasts lacking the *GAP3/DIP5/GAI1* transporters inside cultured macrophages. Compared to wild-type *Histoplasma* yeasts, permease-deficient yeast were not attenuated in their ability to grow intracellularly (Figure 6a). This full proliferation in the absence of amino acid transport led to similar killing of cultured macrophages by *Histoplasma* yeasts (Figure 6b). As a further test of the contribution of amino acid import to *Histoplasma* virulence, the triple-transporter-deficient yeasts were tested for their ability to cause respiratory infections in mice. Since *Histoplasma* yeasts lacking gluconeogenic metabolism are known to be severely attenuated by 8 days post-infection [15], the peak of the fungal lung colonization, the murine infection of transporter-deficient yeasts was also examined at this time point. At 8 days post-infection, no difference was observed in the lung fungal burdens between wild type *Histoplasma* and the triple amino acid transporter-deficient strain (Figure 6c). There was a small but statistically significant increase in spleen fungal burdens in the permease-deficient strain compared to wild type yeast at 8 days post infection. Taken together, these results indicate the Gap3, Dip5, and Gai1 amino acid transporters are dispensable for *Histoplasma* proliferation in macrophages and virulence *in vivo*, suggesting metabolism of an intracellular nutritional source other than free amino acids.

(a-b) Cultured transgenic P388D1 LacZ-expressing macrophages were inoculated with either wild type (“WT,” black circles) or amino acid permease-deficient (“*gap3 dip5 gai1*,” red squares) *Histoplasma* yeasts. (a) At 24 and 48 hours post-infection, macrophages were lysed and intracellular yeast were quantified by enumerating colony forming units (CFU). (b) At 6, 7, and 8 days post-infection, macrophage viability was quantified using the remaining LacZ activity relative to uninfected controls to indicate *Histoplasma* lysis of macrophages. (c) Respiratory infections of C57BL/6 mice indicate *in vivo* virulence. Mice were infected intranasally with wild type (“WT,” black circles) or amino acid permease-deficient (“*gap3 dip5 gai1*,” red squares) *Histoplasma* yeasts. After 8 days post-infection, the fungal burdens in mouse lungs and spleens were quantified by plating of tissue homogenates. Data is plotted relative to the inoculum. In (a), points represent biological replicates. In (b), error bars represent standard deviation among 3 biological replicates. In (c), points represent CFU counts from individual mice. Asterisks denote statistically significant differences in growth as determined via Student’s t test (*, *p* < 0.05; n.s., not significant).

## Discussion

*H. capsulatum* readily metabolizes amino acids for growth, especially as a nitrogen source. For carbon, however, *Histoplasma* yeasts show a clear growth preference for a subset of amino acids (at least when grown *in vitro*). *Histoplasma* expression of multiple amino acid transporters with broad substrate specificities (e.g. Gai1 and Dip5 transporters) suggests lack of transport of any individual amino acid is not responsible for the restricted set of amino acids as nutritional sources. Instead, the limited set of amino acids that provide carbon most likely arises from differences in catabolic pathway efficiency or differential regulation of amino acid catabolic pathways in *Histoplasma* yeasts. Of the 28 transporters identified in this study, those of the 2.A.3.10 family were the most highly expressed and two (*GAP3* and *GAI1*) were particularly induced in yeasts compared to avirulent mycelia. Strikingly, the pattern of amino acid transporter expression by yeasts within macrophages largely resembled that of yeasts in an amino acid-rich environment (Figure 3(a)), not of an amino acid-limited environment. This suggests that nutritionally, the *Histoplasma*-containing phagosome is characterized by abundant amino acids, or alternatively the lack of non-amino acid substrates (e.g. hexoses), which is consistent with observations in other intracellular fungal pathogens [13,14].

While the amino acid transporter expression profiling suggests the phagosome contains amino acids to support *Histoplasma* metabolism, the identities of these amino acids and their intraphagosomal concentrations remain unknown. The full virulence of *Histoplasma* yeasts auxotrophic for tyrosine and phenylalanine demonstrates that the macrophage phagosome has sufficient tyrosine and phenylalanine to rescue these auxotrophies. However, the phagosome lacks sufficient tryptophan as tryptophan auxotrophs have attenuated growth in macrophages [11]. Thus, while *Histoplasma* has the necessary transporters to import amino acids during infection, the phagosome environment does not contain all amino acids in sufficient quantities to meet *Histoplasma’s* nutritional requirements.

Despite multiple lines of evidence indicating the phagosome environment has amino acids and that *Histoplasma* catabolizes amino acids during infection, preventing uptake of amino acids by depletion of the three major amino acid transporters does not attenuate *Histoplasma* virulence. The broad specificity for the amino acids transported by the Gai1, Gap3, and Dip5 transporters indicates a high degree of redundancy, which required simultaneous depletion of all three permeases to sufficiently block amino acid import. In contrast to the nearly 80% reduction in growth rate when grown on amino acids *in vitro*, the amino acid transporter-deficient strain showed full rates of proliferation within macrophages and was fully virulent *in vivo*, indicating amino acid import by these genes is not necessary for intracellular proliferation. It is possible that one of the other putative permeases identified could facilitate amino acid uptake within the phagosome. However, the expression of transporters other than Gai1, Gap3, and Dip5 is minimal, even during residence within macrophages. Furthermore, the capacity of *Histoplasma*’s other permeases to transport amino acids is very low and could not support growth on amino acids in the absence of Gai1, Gap3, and Dip5 when grown *in vitro*. An enticing alternative explanation is that small peptides, rather than free amino acids, are the source of amino acids during macrophage infection. Given that the phagosome harbors lysosomal proteolytic enzymes, such as cathepsins, the phagosome may harbor abundant proteolytic products. Thus, we hypothesize that host proteinases convert proteins into peptides, which then serve as the major nutritional carbon and/or nitrogen substrate(s) for *Histoplasma* yeasts inside the phagosome. Consistent with this, the strain of *H. capsulatum* used in this study cannot grow on undigested protein, but digestion of the protein into peptides as the carbon source can support growth of *Histoplasma* yeasts, including yeasts that lack the ability to import amino acids. Thus, *Histoplasma*’s success as an intracellular pathogen of phagocytes may result from exploitation of the proteolytic nature of the phagosome to provide peptides as a source of amino acids for metabolism to support intracellular yeast proliferation and virulence.

## Supplementary Material

Table S2.docx

Table S1.docx

## Data Availability

Raw data are available via Figshare (https://doi.org/10.6084/m9.figshare.27188757).
