# Supplementary material for: Three transporters, including the novel Gai1 permease, drive amino acid uptake in Histoplasma yeasts
Source: Virulence. 2024 Dec 9;15(1):2438750. doi: 10.1080/21505594.2024.2438750 (PMC11633205; doi:10.1080/21505594.2024.2438750)
Supplement: Table S2.docx [file KVIR_A_2438750_SM2315.docx]

**Table S2. qPCR primers used in this study.**

| Primer | Primer sequence (5' to 3') | Direction^a^ |
| --- | --- | --- |
| 00617-3 | CTTTCCTCCAAGACCCGTTCG | Forward |
| 00617-4 | GGGCCCCATTGCGAAAAGTT | Reverse |
| ACT1-5 | GGTTTCGCTGGCGATGATGCTC | Forward |
| ACT1-9 | AAGGACGGCCTGGATGGAGACG | Reverse |
| CAN1-3 | TCTCGTCAAGCCCGTGTCATG | Forward |
| CAN1-4 | GAAATTGCTTCGCCGCTACC | Reverse |
| DIP5-1 | CGGCGAAATGGCAGCATGGCTT | Forward |
| DIP5-2 | TCCAAACTCCTGGATTCACCCT | Reverse |
| GAI1-3 | CCGGCTTCAACAATCTTCTCAGT | Forward |
| GAI1-4 | CGGGCTGGAGAAGTAGCGGC | Reverse |
| GAP1-1 | TTGGTGTTCGTGGGTATGGGGA | Forward |
| GAP1-2 | GAAGGCGAAGGCAGCAGTAACA | Reverse |
| GAP2-4 | CTTCGCTGCCGCCTTTGGAG | Forward |
| GAP2-5 | GCCAACCCTGACCCTTCCAT | Reverse |
| GAP3-1 | TGCCGTGCTTGCCACCTCTGTT | Forward |
| GAP3-2 | AGGGGAGAAGATGCTCCAGGTT | Reverse |
| HNM1-1 | CTTCACCTCTGCTAGTGTTGCG | Forward |
| HNM1-2 | TTGTCATTGCGACTTTAGGGAG | Reverse |
| PUT4-1 | GTGTCTTGGTGAAATGGCAACT | Forward |
| PUT4-2 | GAGAGCAGCGGCAGCAATTTCG | Reverse |
| RPS15-3 | GGCAGCATCATCGGTATCTACTC | Forward |
| RPS15-4 | TGACGGGTTTGTATGAGATCG | Reverse |
| TEF1-8 | GCTCTGCTTGCTTTCACCCTTG | Forward |
| TEF1-9 | TCTCCTTGTTCCAGCCCTTGT | Reverse |

^a^ Direction relative to gene transcription
