# Supplementary material for: Three transporters, including the novel Gai1 permease, drive amino acid uptake in Histoplasma yeasts
Source: Virulence. 2024 Dec 9;15(1):2438750. doi: 10.1080/21505594.2024.2438750 (PMC11633205; doi:10.1080/21505594.2024.2438750)
Supplement: Table S1.docx [file KVIR_A_2438750_SM2314.docx]

**Table S1A. *Histoplasma* strains utilized in this study.**

| **Strain^a^** | **Genotype^b^** |
| --- | --- |
| G217B | wild type clinical isolate (ATCC 26032) |
| OSU546 | *G217B ura5-42Δ gap3-1 (-8 fs @ nt 25) zzz::pAG21 (G418^R^,GFP) zzz::pED02 (URA5, gfp-RNAi)* |
| OSU547 | *G217B ura5-42Δ gap3-1 (-8 fs @ nt 25) zzz::pAG21 (G418^R^,GFP) zzz:pSR31 (URA5, gfp:GAI1:DIP5-RNAi)* |
| OSU601 | *G217B ura5-42Δ zzz::pAG21 (G418^R^,GFP) zzz::pED02 (URA5, gfp-RNAi)* |
| OSU602 | *G217B ura5-42Δ zzz::pAG21 (G418^R^,GFP) zzz:pSR31 (URA5, gfp:GAI1:DIP5-RNAi)* |

^a^ OSU546, OSU547, OSU601, and OSU602 were derived from OSU194 (Garfoot AL et al., 2016)

^b^ gene designations:

*DIP5:* dicarboxylic amino acid permease

*GAI1:* general amino acid importer

*GAP3:* general amino acid permease

*GFP*: green fluorescence protein

*URA5*: orotate phosphoribosyltransferase

**Table S1B. *Saccharomyces cerevisiae* heterologous expression plasmids.**

| **Plasmid^a^** | **Coding sequence** |
| --- | --- |
| pDR196^b^ | Empty vector |
| pSR09 | Histo217_00617 |
| pSR10 | *H. capsulatum GAP1* |
| pSR11 | *H. capsulatum GAP2* |
| pSR12 | *H. capsulatum GAP3* |
| pSR13 | *H. capsulatum DIP5* |
| pSR14 | *H. capsulatum PUT4* |
| pSR15 | *S. cerevisiae DIP5* |
| pSR17 | *H. capsulatum CAN1* |
| pSR20 | *H. capsulatum GAI1* |
| pSR52 | *H. capsulatum HNM1* |
| pSR60 | *H. capsulatum HXT3* |

^a^ All constructs contained the *URA3* gene for selection. Coding sequences were driven by the *S. cerevisiae PMA1* promoter.

^b^(Meyer A et al., 2006)
